# Contact guidance is cell cycle-dependent

**DOI:** 10.1063/1.5026419

**Published:** 2018-05-30

**Authors:** Kamyar Esmaeili Pourfarhangi, Edgar Cardenas De La Hoz, Andrew R. Cohen, Bojana Gligorijevic

**Affiliations:** 1 Bioengineering department, College of Engineering, Temple University, Philadelphia, Pennsylvania 19122, USA; 2 Department of Electrical and Computer Engineering, College of Engineering, Drexel University, Philadelphia, Pennsylvania 19104, USA; 3 Cancer Biology Program, Fox Chase Cancer Center, Philadelphia, Pennsylvania 19111, USA

## Abstract

Cancer cell migration is essential for metastasis, during which cancer cells move through the tumor and reach the blood vessels. *In vivo*, cancer cells are exposed to contact guidance and chemotactic cues. Depending on the strength of such cues, cells will migrate in a random or directed manner. While similar cues may also stimulate cell proliferation, it is not clear whether cell cycle progression affects migration of cancer cells and whether this effect is different in random versus directed migration. In this study, we tested the effect of cell cycle progression on contact guided migration in 2D and 3D environments, in the breast carcinoma cell line, FUCCI-MDA-MB-231. The results were quantified from live cell microscopy images using the open source lineage editing and validation image analysis tools (LEVER). In 2D, cells were placed inside 10 *μ*m-wide microchannels to stimulate contact guidance, with or without an additional chemotactic gradient of the soluble epidermal growth factor. In 3D, contact guidance was modeled by aligned collagen fibers. In both 2D and 3D, contact guidance was cell cycle-dependent, while the addition of the chemoattractant gradient in 2D increased cell velocity and persistence in directionally migrating cells, regardless of their cell cycle phases. In both 2D and 3D contact guidance, cells in the G1 phase of the cell cycle outperformed cells in the S/G2 phase in terms of migration persistence and instantaneous velocity. These data suggest that in the presence of contact guidance cues *in vivo*, breast carcinoma cells in the G1 phase of the cell cycle may be more efficient in reaching the neighboring vasculature.

## INTRODUCTION

Cancer is one of the leading causes of deaths globally. Cancer mortality is tightly linked to cell migration and metastasis.^1^ During metastasis, tumor cells invade and migrate through the stromal tissue, disseminate via the lymphatic or vascular systems, and colonize distant organs.^2^ While migrating through the tissue, tumor cells are often exposed to guidance cues, resulting in directed migration that facilitates persistent navigation through the tissue and efficient arrival to the lymphatic or blood vessels.^3^ Cues guiding directed migration can be biochemical or biophysical. The best studied biochemical cues are gradients of growth factors that induce chemotaxis.^4^ A number of biophysical cues have been identified by recent studies of extracellular matrix (ECM) properties, one example of which is alignment of collagen fibers, shown to stimulate contact guidance.^5,6^

Cell migration is composed of four interdependent molecular steps that make up the cell motility cycle.^7^ The first step involves formation of adhesive protrusions at the leading edge, whose direction is determined by cell polarization.^3^ The next steps include formation of new adhesions at the cell front, elevation of actomyosin contractility in the cell body, and retraction of the adhesions at the cell rear. Cues that induce directed migration, such as growth factor gradients or aligned collagen fibers, cause local activation of certain Rho family GTPases that are part of the polarity signaling machinery of the cells.^6^ Hence, cells are persistently polarized in the direction of the guiding cues, and therefore, new adhesive protrusions are formed in the same direction, resulting in a guided migration.^8,9^ A consequence of directional migration is an increase in migration persistence, which is also manifested in higher average speed of directionally migrating cells. In the absence of directional cues, cells proceed through the same 4-step motility cycle but migrate randomly.

Based on the established hallmarks of cancer,^10^ it is expected that motile cancer cells actively proliferate. Consistently, motile cancer cell populations have been shown to have gene expression profiles shared with highly proliferative cell populations.^11^ However, at the single cell level, it is unlikely that cells actively engaged in actin reorganization during migration can simultaneously proceed through the cell cycle and cell division due to both structural and energy constraints. To explain how the switch between proliferation and migration may occur at a single cell level, the Go-or-Grow hypothesis was proposed, suggesting a temporal exclusivity in division and migration.^12^ However, assessing this hypothesis was mainly done as ensemble measurements and, so far, has resulted in contradicting reports.^13–16^ For example, in glioma and cervical cancer cell lines, cells in G1 and S phases demonstrate higher speed of 2D random migration than cells in G2.^17^ Melanoma cells in 3D spheroids are shown to possess lower motility in the G1 phase compared to the S/G2 phase, while colon cancer cells *in vivo* are shown to migrate faster in the S/G2/M phase compared to the G1 phase.^18^ Most recently, S/G2 cycling mammary epithelial cells were demonstrated to migrate faster in the wound healing assay compared to G1 cells.^19^ While the current literature suggests that the cell cycle phase influences the cell speed and the extent of cell migration, we hypothesized that the ensemble measurements were preventing a consistent result and thus a rigorous test of the Go-or-Grow hypothesis. Therefore, we conducted a systematic study at the single cell level, where both the cell cycle phase and migration of individual cells could be simultaneously monitored and quantified using automated computational image analysis tools.

Cancer cell migration *in vivo* is mostly directional and guided by contact guidance and/or chemotaxis. In this study, we tested the cell cycle dependency of migration persistence and cell instantaneous velocity in directed or random migration, in both 2D and more physiologically relevant, 3D models. To monitor the cell cycle status in real time, we used nuclear labeling by the FUCCI (Fluorescent Ubiquitin Cell Cycle Indicator) set,^20^ which allows us to distinguish the G1 phase of the cell cycle from the S/G2 phase. Using LEVER (lineage editing and validation)^21–25^ and MAT (multitemporal association tracking)^26,27^ algorithms for computational image analysis, we were able to automate the segmentation, tracking, and analysis of migrating cells. Our results indicate that in both 2D and 3D conditions, directed, but not random, migration is affected by the cell cycle phase. While no cell cycle dependency was observed for either persistence or instantaneous velocity in randomly migrating cells, those engaged in contact guidance were more persistent and faster in both 2D and 3D during the G1 phase of the cell cycle. In the 2D model, addition of a chemotactic gradient increased the speed and persistency of all cells regardless of their cell cycle phase. Taken together, our results suggest that cells in the G1 phase of the cell cycle excel in contact guidance.

## RESULTS

### Establishment and characterization of a 2D model for contact guidance and chemotaxis

**FIG. 1.**
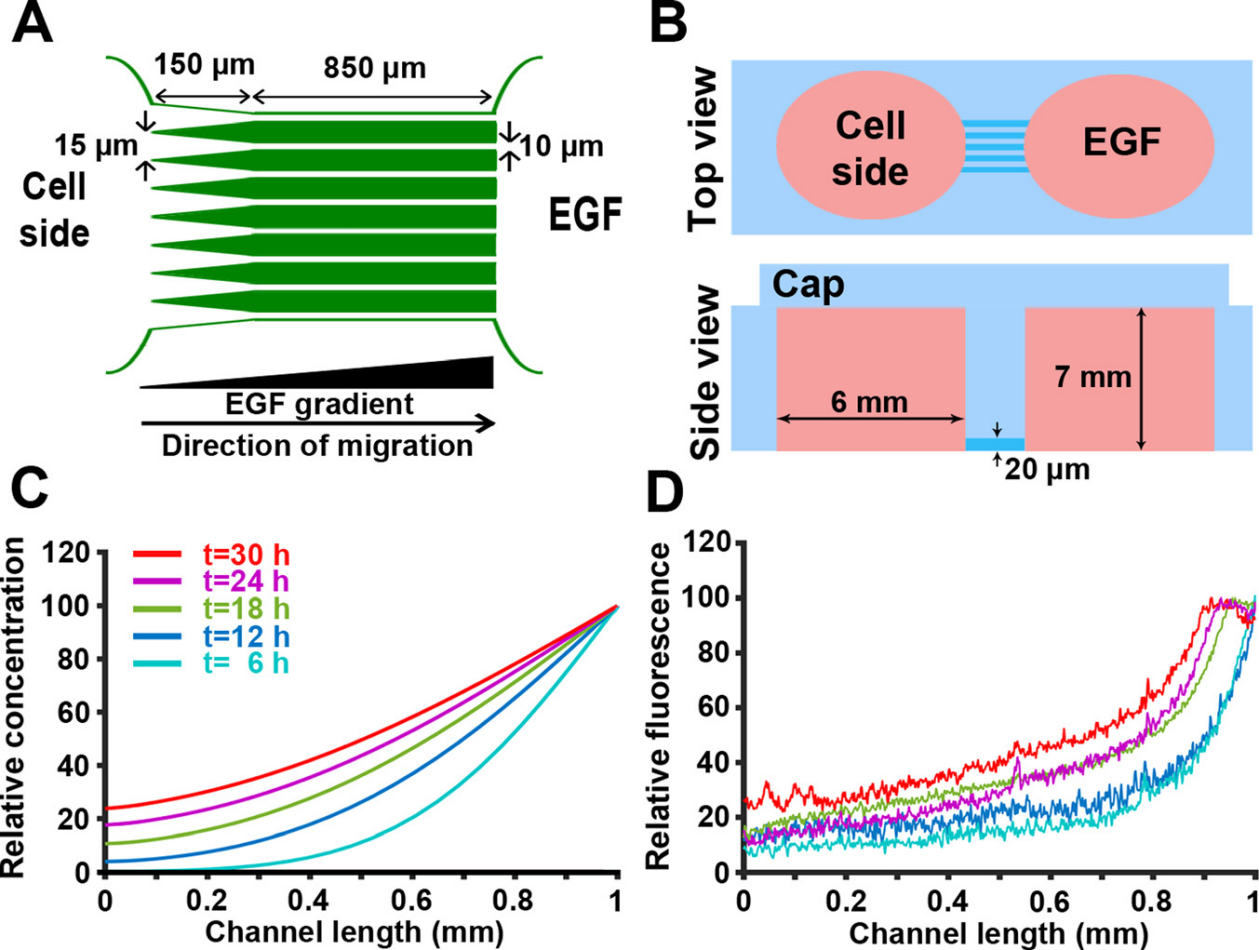
Development and characterization of a 2D model for directed cancer cell migration using a chemoattractant gradient in microchannels. (a) Simplified microchip design. To facilitate the cell entry into 10 *μ*m-wide microchannels, the left end of microchannels was tapered (35 *μ*m width). (b) Top and side views of the microchip; reservoirs were generated using a 6 mm-biopsy punch. (c) Mathematical modeling of the non-linear chemoattractant gradient across the microchannels. Example time points ranging from 6 to 30 h are shown. (d) Gradient of AlexaFluor 405 dextran across the microchannel length, measured at different times 6–30 h. Colors correspond to acquisition times indicated in (c).

To model directed cell migration in 2D, we designed a microchip with microchannels, limiting cell migration to the channels and providing contact guidance cues [Fig. 1(a)]. In addition, in this device, chemotaxis can also be stimulated in cells by introducing a chemoattractant (e.g., Epidermal Growth Factor; EGF) gradient through the microchannels.^28,29^ Microchips were fabricated using PDMS (Polydimethylsiloxane) via soft lithography techniques. Figure 1(b) shows the top and side views of the device with reservoirs on each side of a 20-microchannel array (W 10 *μ*m × H 20 *μ*m × L 850 *μ*m). The left reservoir is loaded with cells suspended in culture media, while the right reservoir acts as a chemoattractant source. To facilitate the cell entry into the channels, the left end of the microchannels is tapered (W 35 *μ*m × L 150 *μ*m). The 10 *μ*m-wide and 20 *μ*m-tall microchannels guide migration of MDA-MB-231 cells along the x-axis while allowing contact with both channel walls.^30,31^

To test the microchip ability to provide a stable gradient across its full length, formation and dynamics of the gradient were first mathematically simulated by solving the unsteady-state diffusion equation using a finite element approach. In the mathematical model, we assumed a constant concentration of the chemoattractant (EGF or dextran) in the right reservoir, as the reservoir volume is six orders of magnitude larger compared to the volume of individual microchannels (∼200 mm^3^ for the reservoir and ∼2 × 10^−4^ mm^3^ for a microchannel). Movie S1 and Fig. 1(c) show the results of the mathematical modeling and formation of the gradient across the microchannels. As indicated in Fig. 1(c), the gradient steepness was predicted to be stable from 6- to 24 h post gradient formation.

Next, we experimentally validated the mathematical model by monitoring the diffusion of fluorescently labeled dextran over time. AlexaFluor 405-labeled 10 kDa dextran has a molecular weight and diffusivity similar to those of EGF [MW (Molecular Weight) = 6 kDa and D = 0.5 × 10^−4^ *μ*m^2^/s]. As shown in Fig. 1(b), mass transfer was limited to the molecular diffusion by capping reservoirs with PDMS pieces, equilibrating the pressure across the system and reducing convection. Figure 1(d) shows quantification of gradient profiles at different time points. While there is a negligible reduction in the gradient steepness over time, as mathematically shown, the average gradient steepness in the system is stable from 6 to 24 h post gradient formation (<10% difference). Since the chemotaxis of MDA-MB-231 cells is affected by the steepness and concentration of the EGF,^32^ quantification of the cell migration parameters (instantaneous velocity and migration persistence) was only conducted on data acquired from 6 to 24 h post gradient formation. Comparing Figs. 1(c) and 1(d) shows that at the beginning of the experiment, the concentration of dextran in the system is higher than what the modeling predicts. This is mainly due to the convectional dextran transfer through the microchannels during the device loading procedure.

### Contact guidance in 2D is cell cycle-dependent

First, we tested the cell cycle-dependency of cancer cell migration in 2D by acquiring time-lapse recordings of FUCCI-MDA-MB-231 cells (Movie S2). To model 2D random migration, cells were plated on gelatin-coated dishes. In the contact guidance model, cells moved along the gelatin-coated microchannels, in the presence or absence of the chemoattractant gradient (Movie S3).

Cell trajectories and the corresponding cell cycle phase information were extracted from time-lapse movies by image segmentation and tracking via algorithms using the LEVER open-source software tools^21–25^ (see section on Materials and methods) (Movie S4). LEVER uses custom segmentation algorithms written in MATLAB combined with the multi-temporal association tracking algorithm (MAT)^26,27^ to quantify the cellular location and morphology in each image frame and to extract the time-course of FUCCI signal intensities for each cell in each image sequence. Extracted data were further used to calculate cell persistence and instantaneous velocity.

**FIG. 2.**
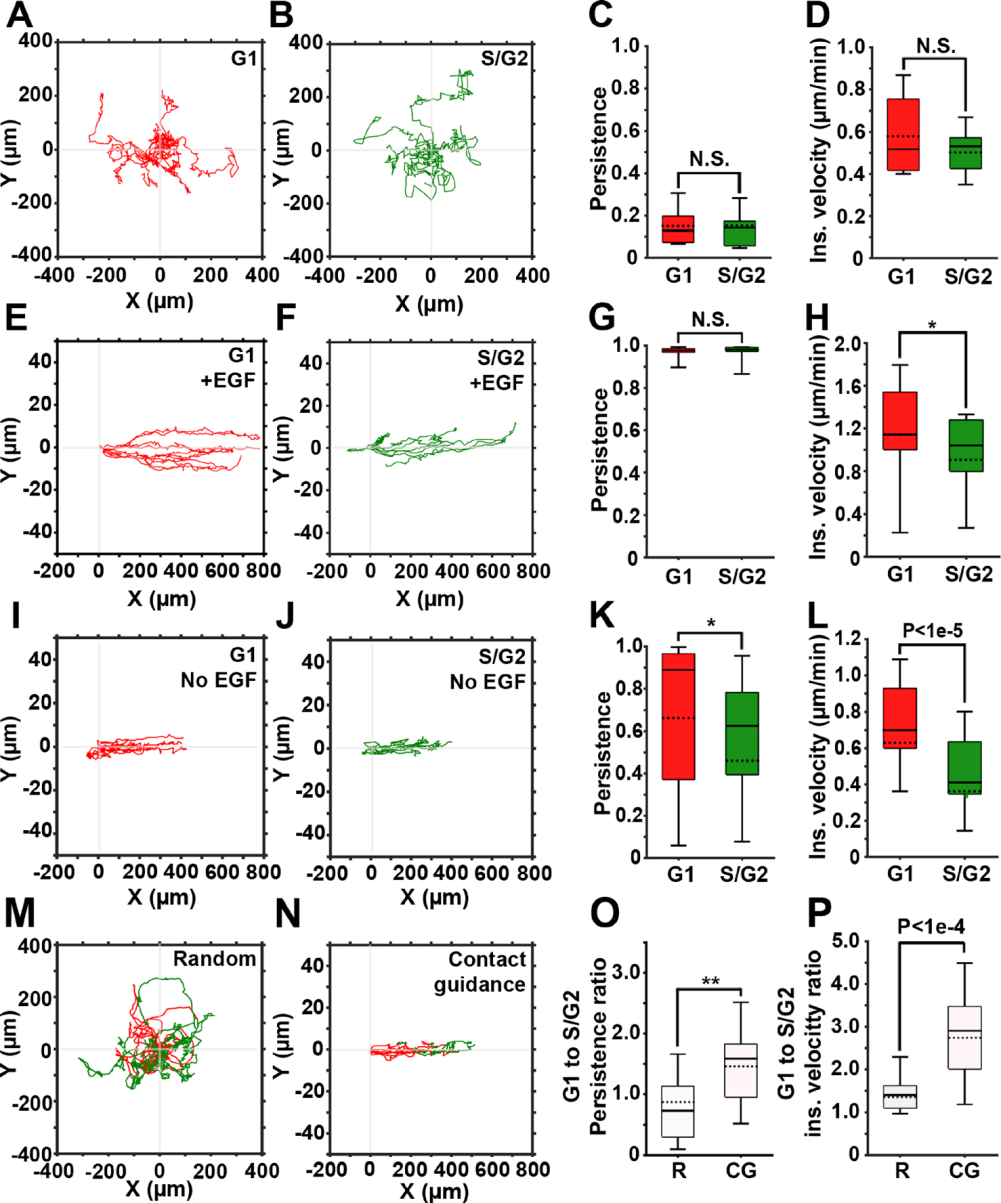
Contact guidance in 2D is cell cycle-dependent. (a) and (b) Representative trajectories of cells in G1 (red) and S/G2 (green) phases, randomly migrating on gelatin-coated plates. (c) and (d) Persistence and instantaneous velocity of cells shown in (a) and (b). (e) and (f) Representative trajectories of cells in G1 (e) and S/G2 (f) phases of the cell cycle migrating inside the gelatin-coated microchannels in the presence of the EGF gradient. (g) and (h) Persistence and instantaneous velocity of cells shown in (e) and (f). (i) and (j) Representative trajectories of cells in G1 (i) and S/G2 (j) phases of the cell cycle migrating inside the gelatin-coated microchannels, in the absence of the EGF gradient. (k) and (l) Persistence and instantaneous velocity of cells shown in (i) and (j). (m) and (n) Representative trajectories of cells monitored continuously as they migrate in G1and S/G2, either randomly (m) or contact guided, in microchannels (n). (o) and (p) G1 to S/G2 persistence ratio (o) and instantaneous velocity ratio (p) for randomly migrating (R) and contact guided cells (CGs). In all box and whisker plots, solid and dotted lines represent the median and mean, respectively.

Figures 2(a) and 2(b) depict representative trajectories of cells randomly migrating in 2D and the corresponding cell cycle phases (G1, red and S/G2, green). Cells in the early S phase express both red and green fluorophores, generating yellow labeling. In our system, these cells amounted to <10% of the total number of cells and were excluded from the quantification. As expected, without guiding cues, cells freely change their polarization direction, resulting in a very low migration persistence, independent from the cell cycle phase [Fig. 2(c)].^3^ Consequently, the instantaneous velocity of cells in G1 shows no significant difference compared to that of cells in the S/G2 phase [Fig. 2(d)]. Taken together, these findings demonstrate that random migration of cancer cells in 2D is not cell cycle-dependent.

Next, we assessed migration of cells subjected to directional migration in 2D [Figs. 2(e)–2(l)]. Microchannels guide the cell migration along the x-axis by limiting the movement in y- and z-dimensions,^30^ while the non-linear gradient of EGF stimulates chemotaxis in MDA-MB-231 cells.^32^ Simultaneous exposure to contact guidance and gradient of a chemoattractant results in a highly persistent cell migration, similar in both G1 and S/G2 cell cycle phases [Fig. 2(g)]. However, the instantaneous velocity of cells in G1 is significantly higher (approximately 20%) than the instantaneous velocity of cells in the S/G2 phase [Fig. 2(h)].

Cell migration in the microchannels was also tested in the absence of the EGF gradient [Figs. 2(i)–2(l)], with contact guidance as the only guidance cue. In the absence of the chemoattractant, cells often switched their direction, which resulted in shorter trajectories [Figs. 2(i)–2(j)] and in decreased migration persistence [Fig. 2(k)], when compared to cells exposed to the chemoattractant [Fig. 2(e)–2(g)]. Here, the difference in the instantaneous velocities of cells in G1 compared to the S/G2 phase was even more pronounced [Fig. 2(l)]. Collectively, our data suggest that contact guidance of the cancer cells in 2D microchannels is cell cycle-dependently regulated. Also, addition of chemotaxis increases the persistence and instantaneous velocity of cells in both G1 and S/G2 phases.

Finally, we monitored cell migration throughout the cell cycle. This allowed for a direct comparison between the G1 and S/G2 migration parameters within an individual cell. Such an approach eliminated contributions of intercellular heterogeneity and stochastic gene expression. Figures 2(m) and 2(n) depict trajectories of cells migrating randomly [Fig. 2(m)] or in microchannels [Fig. 2(n) and Movie S5] color-coded according to their cell cycle phase. Here, the G1 to S/G2 ratios of persistence [Fig. 2(o)] and instantaneous velocity [Fig. 2(p)] were calculated for individual cells. In random migration, ratios are close to 1, indicating no change in cell migration when cells transition from the G1 to the S/G2 phase. In cells within microchannels, ratios are significantly higher, indicating that during contact guidance, cells are both faster and more persistent in the G1 phase compared to the S/G2 phase.

### Contact guidance in 3D is cell cycle-dependent

A number of studies demonstrated that cell migration in 2D may not reflect cell behaviors in more physiological relevant 3D models, where cells can be exposed to tissue-like confinement^33–35^ and reciprocal cell-matrix interactions.^36^ Collagen gels are one example of such 3D models, which provide fibrillar structures resembling the topography of the native tissue ECM and therefore are superior to the homogeneous 3D gels.^37^ In 3D collagen gels with pore size equal to or larger than the diameter of the cell nucleus, breast cancer cells utilize MMP (Matrix Metalloproteinases)-independent migration. In contrast, MMP-dependent migration is present in environments with smaller pores.^38^ Additionally, directed cell migration along aligned and bundled collagen fibers (contact guidance) was shown to significantly increase persistence,^5^ without affecting the instantaneous velocity of cells.^39^

**FIG. 3.**
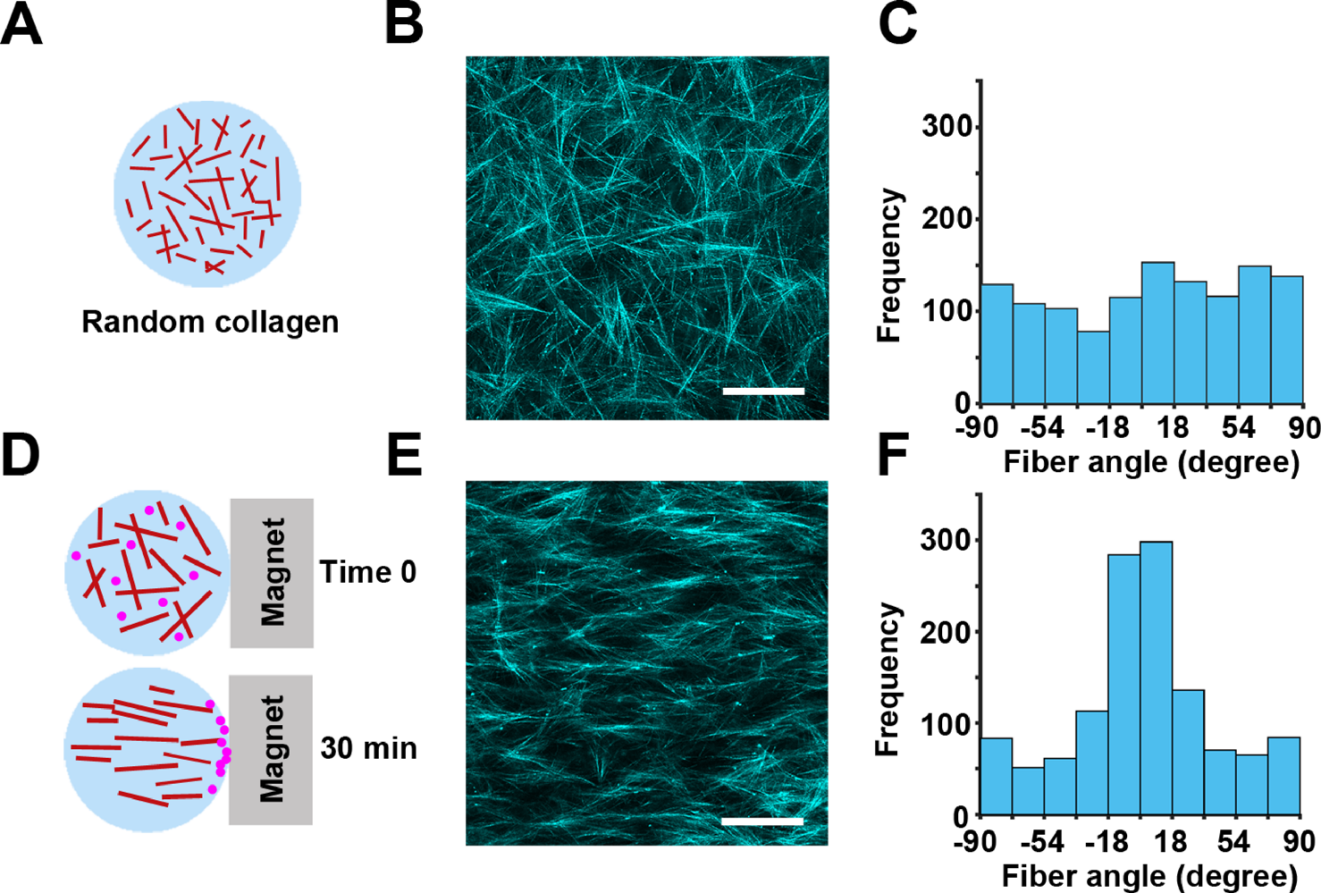
Development of a 3D model for directed cell migration using collagen fiber alignment. (a) Randomly oriented 3D fibrillar collagen was used in the 3D random migration assay. (b) Representative image of the random architecture of collagen fibers imaged by confocal reflection microscopy. (c) Quantification of collagen fiber angles in (b) demonstrating a random distribution of the collagen fiber orientation. (d) Collagen mixed with magnetic beads and exposed to magnetic-induced flow results in 3D collagen with aligned fibers. (e) Aligned architecture of collagen fibers imaged by confocal reflection microscopy. (f) Distribution of collagen fiber angles in (e) shows a Gaussian distribution in the collagen fiber direction, centered at 90° relative to the magnet orientation. The scale bar is 100 *μ*m.

To test the cell cycle-dependency of random and directed cell migration in 3D, we generated collagen gels with either randomly oriented [Figs. 3(a)–3(c)] or aligned fibers [Figs. 3(d)–3(f)]. The aligned fiber architecture was achieved by flowing magnetic beads through the collagen gel.^40^ We confirmed the orientation of the fibers by confocal reflection imaging [Figs. 3(b) and 3(e)] and measuring the distribution of fiber angles in each condition. The randomly oriented fibers show a uniform distribution of fiber angles [Fig. 3(c)], indicating that there is no enrichment of fibers in any particular angle. The aligned fibers show a Gaussian distribution of angles, indicating that the majority of fibers are positioned at the same angle [Fig. 3(f)].

**FIG. 4.**
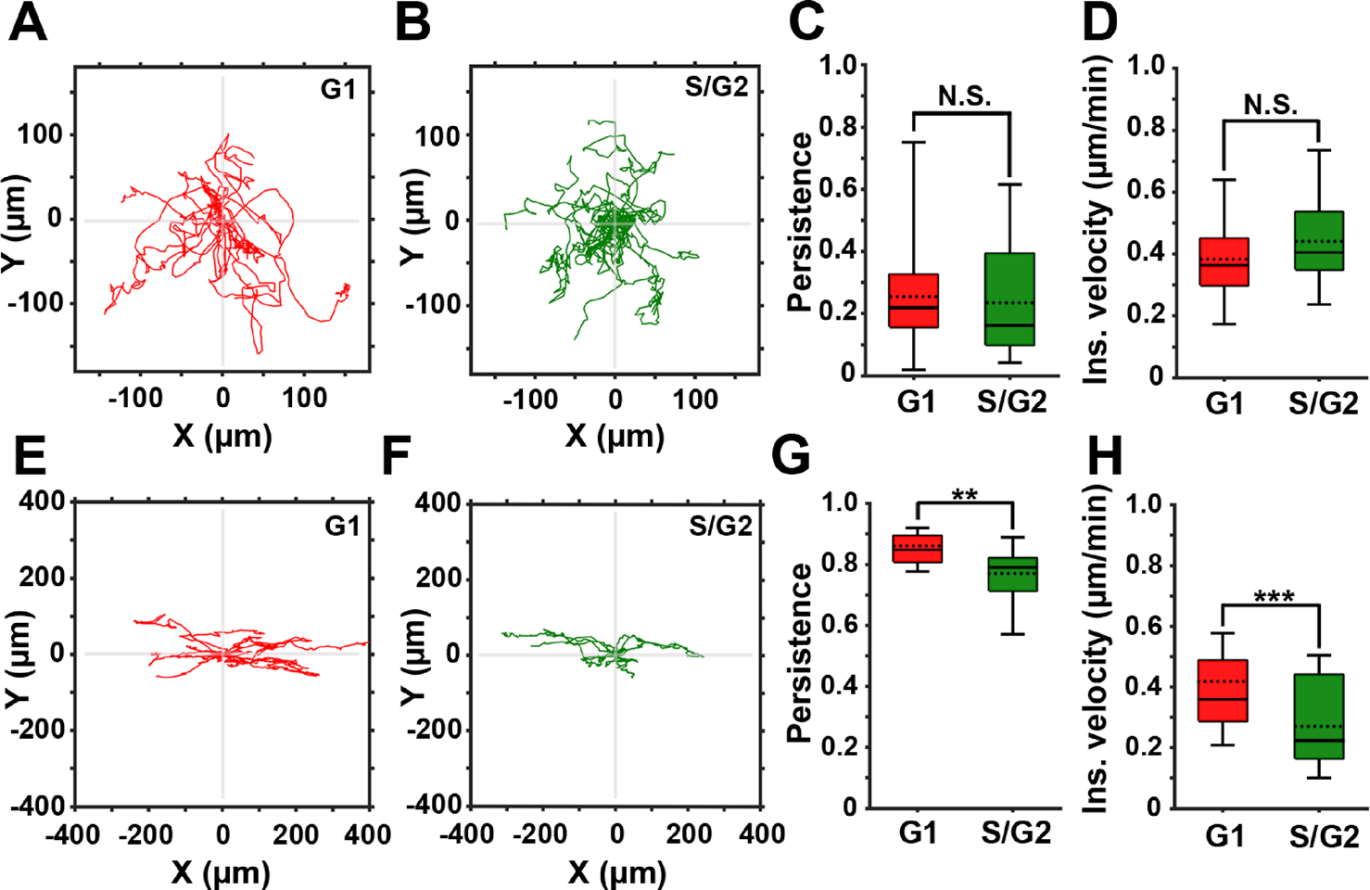
3D contact guidance is cell cycle-dependent. (a) and (b) Representative trajectories of cells in the G1 (red) and the S/G2 (green) phase of the cell cycle migrating in 3D collagen with randomly oriented fibers. (c) and (d) Persistence and instantaneous velocity of cells migrating in 3D collagen with randomly oriented fibers. (e) and (f) Representative trajectories of cells in the G1 (red) and the S/G2 (green) phase of the cell cycle migrating in aligned 3D collagen fibers. (g) and (h) Persistence and instantaneous velocity of cells directionally migrating in aligned 3D collagen fibers. In all box and whisker plots, solid and dotted lines represent the median and mean, respectively.

Using these 3D models, we compared the migration of FUCCI-MDA-MB-231 cells in randomly oriented (Movie S6) or aligned (Movie S7) fibrillar collagen. Time-lapse images were segmented and tracked using LEVER. Consistent with the random migration pattern in 2D, the cell cycle status did not affect persistence nor velocity of cells in randomly oriented fibers in 3D [Figs. 4(a)–4(d)].

In aligned collagen, cell trajectories show higher persistence compared to cells in randomly oriented collagen [Figs. 4(e) and 4(f)]. Furthermore, both cell persistence and velocity are cell cycle-dependent, as quantification of the migration parameters shows that cells in the G1 phase are significantly more persistent and faster than cells in S/G2 [Figs. 4(g) and 4(h)]. In conclusion, our data demonstrate that cells in the G1 phase of the cell cycle exhibit a more persistent and a faster migration in response to directional guidance cues. During random migration, the persistence and velocity of cells in the G1 and S/G2 phases of the cell cycle are similar.

## DISCUSSION

In this study, we sought to determine the relationship between the cell cycle and migration parameters (velocity and persistence) of cancer cells. We established 2D and 3D models of contact guidance and used these models to visualize migration of MDA-MB-231 breast carcinoma cells expressing the FUCCI cell cycle reporter. Using time-lapse live imaging, we demonstrate that cell cycle progression affects the speed of directional but not random cell migration. We show that while exposed to contact guidance cues, cells in G1 outperform those in S/G2 by exhibiting higher persistence and velocity in both 2D and 3D environments.

The extent of the migration, persistence, and velocity of cells can be regulated by extrinsic cues, including ECM properties or chemoattractant gradients,^32,41–43^ and intrinsic cues, such as cell cycle progression. The cross-talk between the cell cycle and motility pathways was suggested by a number of mechanistic reports. For example, integrins and receptor tyrosine kinases were shown to regulate both Rho GTPases, master regulators of cell contractility and actin polymerization,^44^ and G1-related cyclin-dependent kinases.^45^ Last but not least, recently, a direct link between the Aurora A kinase, known to regulate G2/M transition, and the speed of wound healing is suggested.^19^

Our models and method provide a platform for further interrogating the relationship between cell cycle progression and extrinsic cues, and the effects of that interplay not only on cell migration but also on cell invasion. In this regard, a number of studies have started addressing the influence of cell cycle progression over invasion.^46–51^ For example, a recent study in mouse embryonic fibroblasts has shown that the cytoplasmic pool of cyclin-dependent kinase inhibitor p27 is involved in regulating invadosomes and ECM degradation.^52^ On a similar note, anchor cell invasion into vulval epithelium, occurring during development in *Caenorhabditis elegans*, is only performed by cells that are in the G0/G1 phase of the cell cycle.^53^ Finally, a number of *in vivo* tumor studies have reported a negative correlation between tumor growth and invasion, such that tumors which invade and metastasize more efficiently grow slower and vice versa.^54–60^ In melanomas, this phenomenon was described as the *phenotypic switch*.^54^ While no studies, so far, addressed the effect of extrinsic factors on coordination between the cell cycle and invasion, a number of recent works can be used to guide future experiments. For instance, ECM stiffening in mammary epithelial cells was shown to activate cell cycle progression via a FAK (Focal Adhesion Kinase)-Rac-Cyclin D1 pathway,^61^ and applying sheer stress to tumor cells can induce a G2/M arrest through α_v_ß_3_ and ß_1_-mediated pathways,^62^ both of which are likely to affect integrin/FAK-dependent invasion and invadopodia assembly.

Tumor cell arrest during the G1/S transition, by inhibitors of Cdk4/6, is one of the new avenues for breast carcinoma treatment.^63^ While such treatment successfully reduces tumor size, it may also accumulate reactive oxygen species and increase Cyclin D1 expression,^64^ which have been linked to the increase in migration and invasion.^65,66^ Hence, understanding the coordination between the cell cycle and cell migration, as well as the other hallmarks of metastasis, is increasingly important.

## METHODS

### Cell culture and generation of FUCCI-MDA-MB-231

Human breast cancer cell line MDA-MB-231 (ATCC, Manassas, VA) was cultured in DMEM (Dulbecco's Modified Eagle Media) supplemented with 10% FBS (Fetal Bovine Serum) (Atlanta Biologicals, Flowery Branch, GA) and 1% Penicillin/Streptomycin mixture (Gibco, Thermofisher Scientific, Waltham, MA).

Cell line FUCCI-MDA-MB-231^20^ was generated using a self-inactivating lentiviral expression vector system.^67^ Viral particles were produced by co-transfecting mKO_2_-hCdt1 (red) or mAG-hGem (green) with the packaging plasmids (pCAG-HIVgp), G protein of vesicular stomatitis virus (VSV-G), and Rev-expressing plasmid (pRSV-rev) into HEK-293T cells. The supernatant was collected and concentrated using the Lenti-X concentrator (Clontech Laboratories, Inc). High-titer viral solutions were used to co-transduce MDA-MB-231 cells, and top 5% expressors were selected by FACS (Fluorescence-Activated Cell Sorting).

### 2D random migration assay

60 000 FUCCI-MDA-MB-231 cells were cultured on gelatin-coated plates described previously.^68^ Briefly, acid-washed 35-mm glass bottom dishes (MatTek Corporation, Ashland, MA) were incubated with 50 *μ*g/ml poly-l-lysine (Gibco, Thermofisher Scientific, Waltham, MA) for 20 min and then coated with 0.2% gelatin solution for 10 min. Plates were then washed with PBS (Phosphate Buffered Saline) (Gibco, Thermofisher Scientific, Waltham, MA) and cross-linked by 0.2% glutaraldehyde (GTA, Sigma, St. Louis, MO) on ice for 15 min. Next, plates were extensively rinsed with PBS, quenched with 5 mg/ml sodium borohydride (Sigma-Aldrich, St. Louis, MO), and sterilized with 70% ethanol (Decon Laboratories, King of Prussia, PA). Image acquisition was initiated 2 h after cell plating.

### Microchannel fabrication

The microchips containing microchannels were fabricated using soft lithography techniques. First, the microchip design^69^ was created using Autocad (Autodesk, San Rafael, CA) and printed on a glass plate to serve as a photomask. Next, a silicon master was fabricated via spin coating a 4 in silicon wafer (University Wafers, South Boston, MA) with an SU-8 photoresist (MicroChem Corporation, Newton, MA) followed by baking at 70 °C for 20 min, exposing to UV light passing through the photomask transparencies and removing the uncross-linked photoresist by a developer. The silicon master served as a mold for making microchannels, onto which a 10:1 mixture of PDMS:curing agent (Dow Corning, Midland, MI) was poured, degassed under vacuum to remove air bubbles, and cured at 70 °C for 1 h. Next, cured PDMS was carefully peeled from the wafer and cut into single-chip size pieces. Cell- and chemoattractant-side reservoirs were made on each device using a 6-mm biopsy punch. Next, acid-washed 35-mm glass bottom dishes (MatTek Corporation, Ashland, MA) and PDMS devices were activated via oxygen plasma treatment for 30 s at 300 mTorr. Microchips were then assembled by bonding each PDMS device with a MatTek dish. Assembled devices were then sterilized with 70% ethanol followed by repeatedly rinsing the devices with sterile PBS.

### Mathematical modeling of gradient formation within the channels

The dynamics of EGF gradient formation within the microchannels were modeled using MATLAB (Mathworks, Natick, MA) using the partial differential equation toolbox (PDEtool). The geometry of the model included the xy cross-section of a microchannel, and the physics of the model involved a two-dimensional unsteady-state diffusion equation, $\frac{\partial C}{\partial t}=-D(\frac{\partial^{2}C}{\partial x^{2}}+\frac{\partial^{2}C}{\partial y^{2}})$, with a constant-concentration boundary condition applied to the source edge. In addition, a Neumann boundary condition was applied to the sink edge of the channel, indicating that the external flux of the chemoattractant from the system depends on the concentration of the chemoattractant at the sink edge. Finally, no-flux boundary condition was defined for the PDMS walls of the microchannels. The initial concentration of the EGF in the whole system was set to zero. The EGF-water diffusion coefficient, D, was set to 0.5 × 10^−4^ cm^2^/s.

### Experimental validation of gradient formation in the microchannels

Prior to the experiment, sink and source reservoirs of the devices were rinsed and filled with PBS. The PBS in the source reservoir was supplemented with Alexa Fluor 405-labeled 10 kDa dextran (the MW of EGF is 6 kDa), and microchannels were then imaged on a widefield Olympus (Olympus, Tokyo, Japan) microscope for 30 h with 10 min intervals.

The resulting movies of the dynamics of Alexa Fluor 405-dextran diffusion within the microchannels across the two reservoirs were analyzed using Fiji.^70^ Briefly, a “plot profile” was applied to a line drawn through the length of the channels, and fluorescence intensities were recorded at every frame. Relative fluorescence intensities were then calculated via applying the following equation to the raw data:

$$\text{Relative}\;\text{fluorescence}=\;\frac{I\;(x,t_{j})-\;I_{\text{min}}(t_{j})}{I_{max}(t_{j})-\;I_{min}(t_{j})},$$ (1)

where *I*(*x,t_j_*) represents the raw fluorescence intensity recorded at length x of the microchannel and time *t_j_* of the experiment. *I_min_*(*t_j_*) and *I_max_*(*t_j_*) are the minimal and maximal fluorescence intensities recorded in the microchannel at time *t_j_*.

### 2D contact guidance using microchannels

Prior to cell plating, sink and source reservoirs of the microchips were rinsed with PBS and then filled with culture media. 25 000 cells were plated in the cell (sink) reservoir, and the devices were placed in an incubator for 2 h for cell adhesion. Next, the media in the source reservoir was supplemented with 20 *μ*M EGF (Thermofisher Scientific, Waltham, MA), and microchips were imaged with a widefield Olympus (Olympus, Tokyo, Japan) microscope for 30 h with 10 min intervals. Movies were then processed for extracting migration parameters. In the movie processing, cell tracking was initiated upon the cell entry into the tapered section of the microchannels.

### Collagen alignment

Collagen fibers were aligned by incorporating paramagnetic polystyrene beads (PM-20-10; Spherotech, Lake Forest, IL) into a 1.5 mg/ml collagen mixture at 4% (v/v) and exposing the mixture to the magnetic field of a neodymium magnet (BZX0Y0X0-N52; K&J Magnetic, Pipersville, PA) during collagen polymerization.^40,71^ This step was performed at room temperature for 30 min. As schematically shown in Fig. 3(d), magnetic-field induced flow of magnetic beads within the collagen matrix aligns collagen fibers. Collagen alignment was assessed by confocal reflection microscopy followed by image processing by ct-FIRE^72^ to extract fiber angles.

### 3D migration assays

40 000 FUCCI-MDA-MB-231 cells were suspended in 50 *μ*l of a collagen mixture containing the following: 1.5 mg/ml rat tail collagen I (Corning, Tewksbury, MA), 5 *μ*l 10× PBS, 10% FBS (Atlanta Biologicals, Flowery Branch, GA), 1% Penicillin/Streptomycin, DMEM (Gibco, Thermofisher Scientific, Waltham, MA), 4% paramagnetic polystyrene beads (PM-20-10; Spherotech, Lake Forest, IL), and 1 N NaOH. The mixture was vortexed at 4 °C for 5 min and pipetted in a 35-mm glass bottom plate (MatTek Corporation, Ashland, MA). The collagen mixture was polymerized at room temperature for 30 min and formed a 3-mm thick gel. For collagen alignment, this step was conducted by positioning the plate adjacent to a neodymium magnet (BZX0Y0X0-N52; K&J Magnetic, Pipersville, PA). Next, 1 ml DMEM containing 10% FBS and 1% antibiotics was pipetted into the plate. The plate was then imaged via a widefield Olympus IX81 (Olympus, Tokyo, Japan) microscope for 30 h with 10 min intervals.

### Live cell imaging

Live cell imaging was performed via a widefield Olympus (Olympus, Tokyo, Japan) microscope equipped with a LED (Light-emitting diode) lamp, Hamamatsu Orca 16-bit CCD (Hamamatsu, Hamamatsu city, Japan), an automated z-drift compensation IX3-ZDC (Olympus, Tokyo, Japan), an automated Prior stage (Prior Scientific, Rockland, MA), and an environmental chamber. For live cell imaging, a regular culture medium was supplemented with 1:100 Oxyfluor (Oxyrase, Mansfield, OH) and 10 mM sodium lactate (Sigma-Aldrich, St. Louis, MO) to reduce phototoxicity. Time lapse imaging was conducted at a single focal plane using an Olympus 20× 0.7 NA objective, and images were acquired at FITC (Fluorescein Isothiocyanate), TRITC (Tetramethylrhodamine Isothiocyanate), and bright field channels. Images were collected every 10 min for 30 h.

### Computational image analysis

Each image is first segmented and then tracked using the approach previously described for the LEVER software tools.^21–24^ The segmentation processed the two FUCCI channels using a denoising algorithm^25^ that models imaging noise as a combination of slow-varying low frequency background noise and high frequency shot noise. The denoising algorithm uses a Gaussian low-pass filter, with a kernel size equal to 10% of the image size, combined with a median filter with a support of 3 × 3 pixels. The segmentation treats the fluorescence and phase channels separately. The fluorescence images start with an adaptive Otsu thresholding, followed by a connected component analysis. The phase segmentation identifies bright and dark foreground pixels as those falling greater than one standard deviation from the mid-level background pixels. These bright and dark pixels are combined with an Otsu thresholded gradient image to produce the final foreground. The intersection of the phase and fluorescence channel segmentations is used as the final segmentation.

Following segmentation, the images are tracked to establish temporal correspondences among the segmentation results. The MAT^26,27^ algorithm has been widely applied in a number of applications and is used here. MAT uses a minimum spanning tree optimization to solve the data association problem across multiple image frames (here set to three) in polynomial time. The first tracking step is to compute a cost function between segmentations based on differences in the spatial location, shape and size, and fluorescence intensity signals. For each cell, the fluorescent intensity is taken as the mean fluorescent signal within the segmentation boundaries. Cells that are separated by more than twice the maximum radius of either cell or with a size difference of more than 90% between frames are gated or considered to have an infinite tracking cost. The gate is adaptive, with the constraint adjusted upwards until at least five possible tracking matches are obtained. MAT then uses the cost function to compute optimal tracking associations between segmentations. The LEVER program then allows the results to be visualized and optionally for any errors to be corrected manually.

Cell tracks were manually analyzed to correct for missegmented cells and assess instantaneous velocity (displacement between two frames divided by time) and cell persistence (net cell displacement over the course of the experiment divided by the total displacement).

Cells which were tracked and analyzed throughout the cell cycle phases G1 and S/G2 show a transient (∼2 h long) overlay, between G1 (red) and S/G2 (green) fluorescence, shown as yellow (Movie S5). These cells were classified as either G1 (>50% of maximum red fluorescence) or S/G2 (<50% maximum red fluorescence).

Ethics approval was not required for this work.

## STATISTICAL ANALYSIS

The two-tailed Student T-test was performed for all statistical analysis. The statistical significance was defined as *P < 0.05, **P < 0.01, and ***P < 0.001. Data are shown as means ± SEM.

## SUPPLEMENTARY MATERIAL

See supplementary material for Movies S1–S7.
